# Commentary: Dogs and the classic route of Guinea Worm transmission: an evaluation of copepod ingestion

**DOI:** 10.3389/fvets.2020.00404

**Published:** 2020-08-04

**Authors:** M. Teresa Galán-Puchades

**Affiliations:** Parasite and Health Research Group, Department of Pharmacy, Pharmaceutical Technology and Parasitology, Faculty of Pharmacy, University of Valencia, Valencia, Spain

**Keywords:** Guinea worm eradication, dog dracunculiasis, *Dracunculus medinensis*, water-borne transmission, food-borne transmission

Dracunculiasis was largely considered a parasitic disease exclusively affecting humans. That is why all the control measures taken, aimed at its global eradication, were exclusively applied to humans. Currently, Guinea worm disease is considered a zoonosis, with dogs being the main reservoir, reaching high rates of infection, thus jeopardizing its eradication. An alternative route of foodborne parasite transmission has been suggested for dogs by means of the ingestion of infected frogs and/or fish. In addition, a recent study carried out in dogs to assess their ability to ingest copepods while drinking has cast doubts on the key role of drinking water in the dracunculiasis epidemiology. As a result, both routes of transmission, waterborne and foodborne, are discussed.

Around since antiquity, Dracunculiasis, or Guinea worm disease, is a parasitic infection known to affect humans (1). According to the World Health Organization, dracunculiasis was the first parasitic disease set for eradication (https://www.who.int/dracunculiasis/eradication/en/). Hosts become infected by drinking water contaminated with infected copepods harboring the infective L3 larvae. Once infected, the adult female inhabits the host's subcutaneous tissue. A blister is formed on the skin of the host and the parasite embryos are released from the female when the ulcer is exposed to water.

Ever since Fedcheko described the *Dracunculus medinensis* life cycle in 1870 (2) and until 2014, only the waterborne route of Guinea worm transmission was considered in the epidemiology of human dracunculiasis. Guinea worm disease has classically been considered an anthroponosis, i.e., an infectious disease affecting exclusively humans. However, dracunculiasis is nowadays considered a zoonosis since several mammals, mainly dogs, act as reservoirs of the disease in several African countries (3).

In 2014, Eberhard and colleagues, in an attempt to give an explanation for the large number of infected dogs compared to the few scattered human cases in Chad (Africa), proposed a foodborne route of transmission, i.e., by eating aquatic paratenic/transport hosts—frogs and fish, respectively—harboring the infective larvae (4). Considering that the great success of the Guinea worm eradication program (from 3.5 million human cases in the 1980s to only 53 in 2020) (5) has been achieved through the implementation of control measures only against waterborne transmission, the foodborne route seems to be only of anecdotal importance, at least in humans (6).

The classic waterborne route was recently evaluated in dogs by Garret et al. (7). In their experiment, groups of dogs were given drinking water containing different concentrations of copepods of similar size and species to those which can act as intermediate hosts for the infective Guinea worm larvae. The quantity of copepods ingested by the dogs was estimated and the probability of dogs becoming infected with sufficient male and female larvae to establish an infection was determined using field data on the prevalence of this parasite in wild copepods. According to their results, the authors of the study concluded that drinking water may be an unlikely route for dogs (7).

Consequently, the fact that, in accordance with the experiment carried out by Garret and colleagues, the waterborne route of transmission is unlikely for dogs leads to the assumption that the other possible route of acquiring Guinea worm infection, the foodborne route, i.e., by means of eating infected frogs and fish, should be considered to have a higher likelihood in order to explain the high rate of dog dracunculiasis in Chad.

The various likelihoods are discussed below:

*I) Likelihood of eating infected frogs*. To date, eight infective larvae of *D. medinensis* have been found in the muscles of only five frogs out of 364 studied; that is a 1.37% prevalence (8, 9). Although further studies are required to establish the real approach to frog dracunculiasis prevalence, it will probably be not much higher. The existence of infected copepods in ponds is presumed to be relatively low, and the likelihood of dogs ingesting multiple infected copepods during the 2 to 3 weeks in which copepods are infected with L3 may be low (7). Since frogs and dogs share the same waterborne route of dracunculiasis infection (the ingestion of infected copepods), tadpoles or frogs will have an even lower likelihood of becoming infected (considering their small size compared to dogs). We therefore ask the following: how many frogs have to be eaten by a dog to have the chance of ingesting the infected ones (with male and female larvae) to finally be infected? Considering this low likelihood, the frog route probably does not have any epidemiological importance in the transmission. In this sense, a recent paper by McDonald and colleagues determined that the diet of Chadian dogs consisted basically of human feces and potatoes, peanuts, and rice. Other items, including frogs and fish, are apparently infrequently eaten. The authors consider novel Guinea worm routes of infection as occasional introductions (10). In addition, a study conducted in Chad showed that there were no differences in consumption of aquatic animals, including frogs, between human dracunculiasis cases and controls (11).

*II) Likelihood of eating infected fish*. Although L3 larvae have not been found in any fish so far, fish are considered possible transport hosts (8). In experimental infections, after ingesting infected copepods, larvae remained infective in fish intestines. L3 larvae were highly susceptible to desiccation and exhibited a short life span in the fish intestinal tract (8). Therefore, for dogs to become infected, those transport fish should be caught, taken to the village, eviscerated, and have their entrails discarded in order for dogs to eat them (mostly the infected guts among all the non-infected ones), and the entire process must take place fast enough to prevent larvae desiccation/death. According to the Carter Center/CDC, dog dracunculiasis cases increase considerably in summer (12). The incubation period of *D. medinensis* is around 1 year, and, consequently, dogs become more frequently infected in summer when temperatures reach up to 43°C in Chad (in the shade). It is not believed that Chadian fishers are worried about preventing entrails, or discarded small fish, from desiccation. There is consequently not a high likelihood of this fish route to be the main cause.

*III) Likelihood of drinking water with infected copepods*. Dogs drink unsafe (unfiltered) water several times a day every single day of their lives, and this logically continues in large amounts during the summer, precisely when dog infection increases. Dogs are therefore exposed to permanent risk by the water route. The type of food consumed by dogs can vary, but dogs drink water on a daily basis from whatever water source they may find. The lack of any reliable dog surveillance program before 2012 (like the one currently conducted) does not make it possible to establish the exact degree of exceptionality of the dog epidemiology in Chad, where humans, unlike dogs, thanks to the Guinea Worm Eradication Program, have been drinking filtered water—the reason why only sporadic cases have been detected.

According to their findings, Garret and colleagues do not rule out or minimize the waterborne route, but they doubt its epidemiological importance in dog dracunculiasis (7). However, the same authors recognized the limits of their experiment due to, among other factors, the small sample size chosen. Besides, the dogs were kept at 21°C, not precisely at the extreme temperatures in Chad in the peak of the transmission season (40–43°C). Consequently, the drinking habits of dogs could not be comparable. Furthermore, they used copepods from the USA, which may behave differently from those from Chad. However, and more importantly, the authors did not consider that the copepods used in the experiment were non-infected ones. It has been demonstrated that non-infected and infected copepods with helminth larvae, not only with *D. medinensis*, display a different behavior that positively affects parasite transmission (13–16). Therefore, Garret and colleagues' conclusions could be entirely accepted as well as reasonably discussed based on the data currently known.

## Author Contributions

The author confirms being the sole contributor of this work and has approved it for publication.

## Conflict of Interest

The author declares that the research was conducted in the absence of any commercial or financial relationships that could be construed as a potential conflict of interest.
